# Supplementary material for: A Self-Regulated Microfluidic Device with Thermal Bubble Micropumps
Source: Micromachines (Basel). 2022 Sep 28;13(10):1620. doi: 10.3390/mi13101620 (PMC9612009; doi:10.3390/mi13101620)
Supplement: Supplementary file 1 [file micromachines-13-01620-s001.zip › micromachines-1909727-supplementary v3.pdf]

A Self-Regulated Microfluidic Device with Thermal Bubble Micropump

1. Supplementary Analysis

A. Theory and Design Analysis of Micropump

In order to understand and analyze the theory of the micropump, we regarded the microchannel in the micropump as a liquid-filled pipe with a diameter of d and a length of L_T. The liquid inlet is marked as I and the liquid outlet is marked as O, as shown in the following **Figure S1**. When a mobile heat in the microchannel is heated to a sufficient amount of heat q to generate a moving thermal bubble (length Lg), the liquid in the microchannel flows, where the evaporation surface and condensation surface are denoted by E and C said. Because the pressure on the evaporation surface E is higher than that at the outlet O, a pressure gradient is formed to cause the liquid flowing to the outlet. Similarly, the pressure at the condensation surface E is lower than the pressure at the inlet I, producing a pressure gradient from the liquid inlet to the condensation surface E, and forming the liquid flowing from the inlet to the condensation surface C. When the heat source moves from the inlet of the microchannel to the outlet, the fluid arises a net displacement of. The physical model of the theoretical analysis of the micropump is shown in **Figure S1a**. The pumping mechanism described above can be practically achieved using the sequential actuation of bubbles generated by several heaters distributed along the channel. To illustrate, we give an example, **Figure S1b** shows the moving steam sections produced by three heaters sequentially. It can be seen that as the heater is gradually turned on and off, steam is formed and collapsed in the channel to achieve the desired pressure gradient.

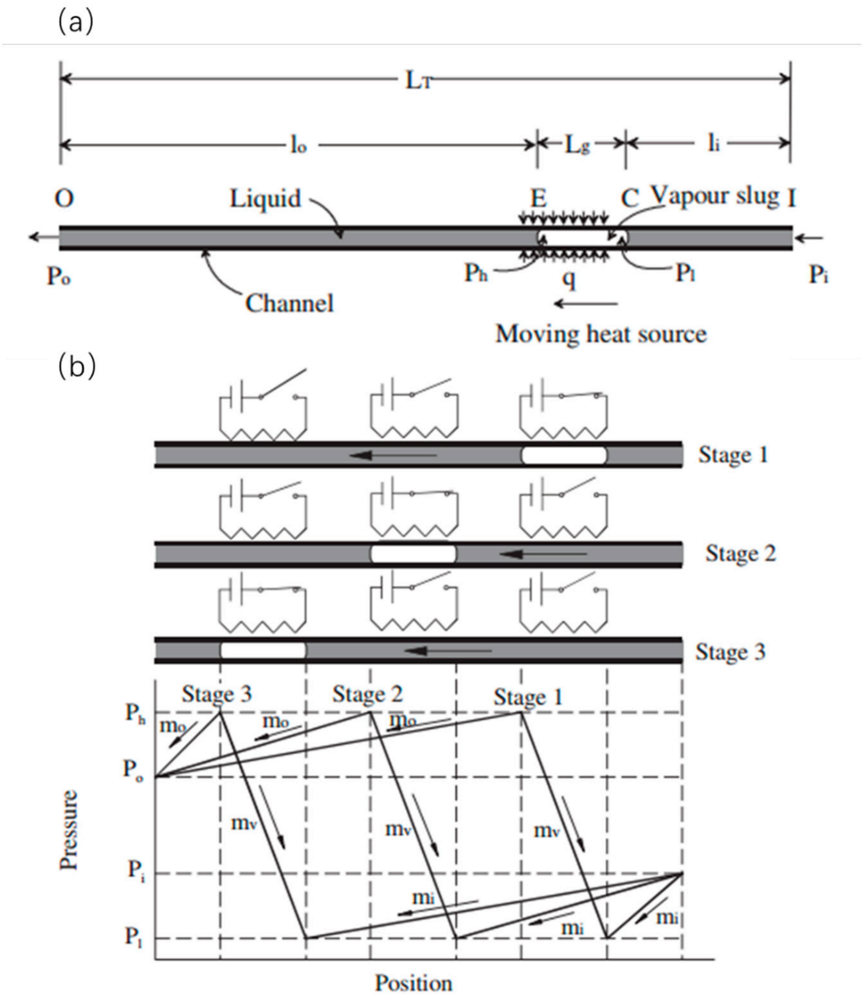

Figure S1. The analysis about fluid flow. **(a)** Physical model for theoretical analysis of the micropump. **(b)** Description of the principle of the micropump.

We need to analyze the pumping mechanism approximately. It is known that for the laminar flow theory of an incompressible viscous liquid in a circular channel, the relationship between pressure drop and velocity can be obtained from the Hagen-Poiseuille equation:

$$-\Delta p = \frac{32\mu L u}{d^2}$$

The expression can also be:

$$u = \frac{d^2(-\Delta p)}{32\mu L}$$

Where d is the pipe diameter, L is the fluid propagation distance, and μ is the fluid viscosity. The fluid velocity at point E is the result of overall motion of liquid in the OE section combined with evaporation of liquid at point E.

$$\frac{dl}{dt} = u + u_e$$

Where l is the distance between point E and point i. Evaporation of fluid caused the moving speed of point E which can be obtained by assuming that the applied heating power is entirely used for evaporation:

$$u_e = \frac{4}{\pi}\frac{q}{\gamma\rho d^2}$$

Where q is the heating power, γ is the heat of evaporation, and ρ is the liquid density. therefore:

$$\frac{dl}{dt} = \frac{d^2(-\Delta p)}{32\mu l_0} + u_e$$

Where $-\Delta P = P_h - P_0$, l_0 is the distance from point O to E, $l = L_T - l_0$. Let $A = d^2(-\Delta P)/(32\mu)$, the above formula can be written as:

$$dt = \frac{L_T - l}{(A + u_e L_T) - u_e l} dl$$

Given the initial condition:

$$\begin{cases} t = 0 & l = 0 \\ t = T_0 & l = L_T \end{cases}$$

Solve the equation:

$$T_0 = \frac{L_T}{u_e} + \frac{A}{u_e^2}\ln\left(\frac{A}{A + u_e L_T}\right)$$

Where T_0 is the time of evaporation surface E from inlet to outlet. Then the mass flow out at outlet O:

$$m_0 = \frac{dM_0}{dt} = \frac{\pi d^4 \rho(-\Delta P)}{128\mu l_0}$$

Notice: $l_0 = L_T - l$, and

$$dt = \frac{L_T - l}{(A + u_e L_T) - u_e l} dl$$

then:

$$dM_0 = \frac{B}{(A + u_e L_T) - u_e l} dl$$

Where M_0 is the total mass flow rate in the time from 0 to t, denoted by B:

$$B = \frac{\pi d^4 \rho(-\Delta P)}{128\mu}$$

For the integral equation of the total mass flow rate, there are boundary conditions:

$$\begin{cases} l = 0 & M_0 = 0 \\ l = L_T & M_0 = M_0 \end{cases}$$

Integrating the equation we can get:

$$M_0 = \frac{B}{u_e}\ln\left(\frac{A + u_e L_T}{A}\right)$$

In T_0 time, The average mass flow of the evaporation surface E to flow from inlet to outlet is given by:

$$\overline{m}_0 = \frac{M_0}{T_0} = \frac{B\ln((A + u_e L_T)/A)}{L_T + (A/u_e)\ln(A/(A + u_e L_T))}$$

Similarly, the time Ti taken for the condensing surface C to move from the inlet, the total amount of liquid M_i entering the channel from time 0 to time t, and the average mass flow over a cycle can be obtained:

$$\overline{m}_i = \frac{D\ln((C + u_c L_T)/C)}{L_T + (C/u_c)\ln(C/(C + u_c L_T))}$$

Where u_c is the moving speed of point C caused by steam condensation:

$$C = \frac{d^2(P_i - P_1)}{32\mu}$$

$$D = \frac{\pi d^4 \rho (P_i - P_1)}{128\mu}$$

Micropump in Periodic Steady State:

$$\begin{cases} T_0 = T_i \\ u_e = u_c \\ M_0 = M_i \\ \overline{m}_0 = \overline{m}_i = m \end{cases}$$

Where m is the mass flow rate of the micropump, so:

$$\begin{cases} Aln\left(\frac{A}{A + u_e L_T}\right) = Cln\left(\frac{C}{C + u_c L_T}\right) \\ Bln\left(\frac{A}{A + u_e L_T}\right) = Dln\left(\frac{C}{C + u_c L_T}\right) \end{cases}$$

Then:

$$P_h - P_0 = \frac{m[L_T + (A/u_e)ln(A/(A + u_e L_T))]}{(\pi d^4 \rho/128\mu)\ln((A + u_e L_T)/A)}$$

And:

$$P_i - P_1 = \frac{m[L_T + (C/u_c)ln(C/(C + u_c L_T))]}{(\pi d^4 \rho/128\mu)\ln((C + u_c L_T)/C)}$$

For the evaporation surface mass flow:

$$m_{ec} \frac{\pi d^4 \rho_v (P_h - P_1)}{128\mu_v L_g} = \frac{q}{\gamma}$$

Where ρ_v and μ_v are the density and viscosity of the steam respectively. Therefore:

$$P_h - P_1 = \frac{qL_g/\gamma}{(\pi d^4 \rho_v/128\mu_v)}$$

Combining the above equations to obtain the relationship between the pressure difference (P_0-P_i) and the mass flow(m) of the micropump under the design and operation:

$$P_0 - P_i = \left(\frac{qL_g k_v}{\gamma} - mk\left(\frac{P_h - P_1}{P_i - P_1}\right) \times \left[\frac{L_T}{ln\left(1 + \frac{128kqL_T}{\pi d^4 \gamma (P_i - P_1)}\right)} - \frac{\pi d^4 \gamma (P_i - P_1)}{128kq}\right]\right)$$

$$\times \left(\frac{\pi d^4}{128} + mk\frac{1}{(P_i - P_1)} \times \left[\frac{L_T}{ln\left(1 + \frac{128kqL_T}{\pi d^4 \gamma (P_i - P_1)}\right)} - \frac{\pi d^4 \gamma (P_i - P_1)}{128kq}\right]\right)^{-1}$$

Where $k=\mu/\rho$, $k_v=\mu_v/\rho_v$. From this, the relationship between the flow Q and the head H of the micropump can be obtained:

$$H=\left(\frac{qL_g k_v}{\gamma\rho g}-\frac{Qk}{g}\left(\frac{P_h-P_1}{P_i-P_1}\right)\times\left[\frac{L_T}{ln\left(1+\frac{128kqL_T}{\pi d^4\gamma(P_i-P_1)}\right)}-\frac{\pi d^4\gamma(P_i-P_1)}{128kq}\right]\right)$$

$$\times\left(\frac{\pi d^4}{128}+\frac{\rho Qk}{(P_i-P_1)}\times\left[\frac{L_T}{ln\left(1+\frac{128kqL_T}{\pi d^4\gamma(P_i-P_1)}\right)}-\frac{\pi d^4\gamma(P_i-P_1)}{128kq}\right]\right)^{-1}$$

Let Q=0, inferred:

$$H_{max}=\frac{128qL_g k_v}{\pi d^4\gamma\rho g}$$

Therefore, the maximum flow at zero head is:

$$Q_{max}=\left(\frac{qL_g k_v}{\gamma\rho k}\left(\frac{P_i-P_1}{P_h-P_1}\right)\right)\times\left(\left[\frac{L_T}{ln\left(1+\frac{128kqL_T}{\pi d^4\gamma(P_i-P_1)}\right)}-\frac{\pi d^4\gamma(P_i-P_1)}{128kq}\right]\right)^{-1}$$

B. Fabrication

The microchip is manufactured by MEMS technology: In this process, the bulk silicon is first covered with a thin layer of silicon dioxide, and then the backside oxide layer is dry-etched to form the inlet and outlet, and the oxide layer is used as a mask layer for anisotropic etching. The photoresist (sacrificial layer material) was spin-coated as a sacrificial layer and patterned, after which a thin parylene film was coated on the front side of the silicon wafer as the top of the channel. A layer of polyimide is spin-coated and patterned to increase the mechanical strength of the channel and prevent channel collapse. Next, the silicon remaining in the etch holes on the back side is removed until the front side silicon oxide layer is etched. After that, the oxide at the bottom of the cavity was put into an HF acid pool to remove. The photoresist (sacrificial layer material) was removed using acetone to create an open channel through the opened access port.

We placed a row of 24 heating films (thermal foaming resistors) on the silicon substrate, with a spacing of 24.3 μm, a diameter of 1 mm through holes, and an edge-to-edge spacing of 2 mm. The lead part of the control signal is placed on the front side, meanwhile, we added a temperature sensor in order to achieve temperature monitoring. **Figure S2** shows the process of forming a micropump channel using MEMS, where resistor placement, lead routing, and sensor setup are omitted.

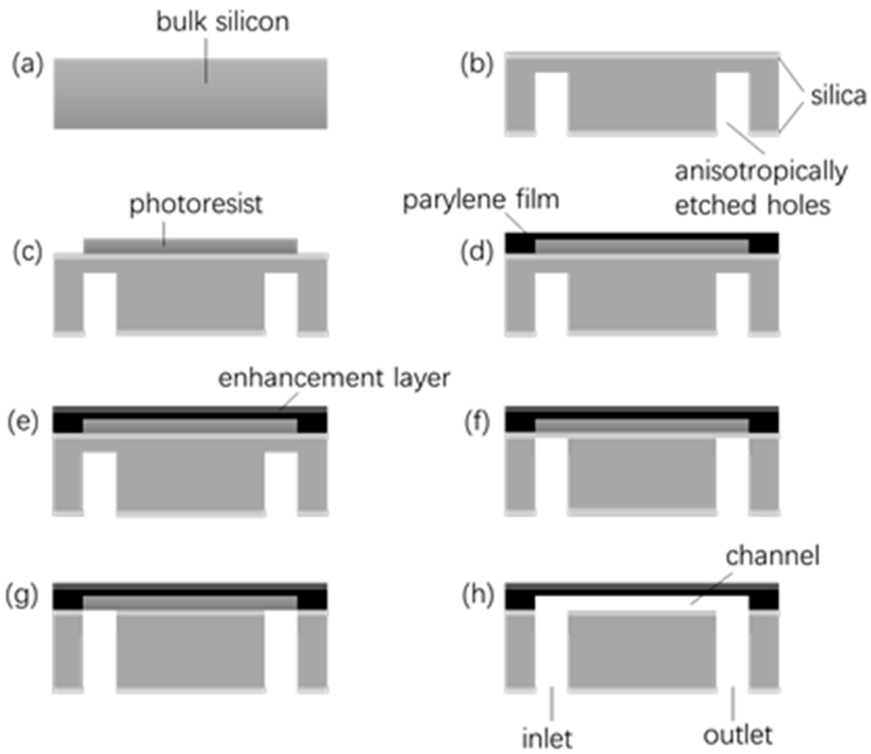

Figure S2. The process of microchannel formation**. (a)** Approximate morphology of bulk silicon. **(b)** The front and back sides are covered with an oxide layer as a mask layer for anisotropic etching. **(c)** spin-coat a layer of photoresist on the front side and pattern. **(d)** Coating a thin parylene on the front side of the silicon wafer as the top of the channel. **(e)** spin-coating a layer of polyimide and pattern to enhance the mechanical strength of the channel and prevent channel from collapsed. **(f)** Remove the silicon remaining in the backside etched hole until the frontside silicon oxide layer is etched. **(g)** Using HF to remove oxide layer at the bottom of the cavity. **(h)** Using acetone or other solvent to remove the photoresist (sacrificial layer).

2.Supplymentary Figure

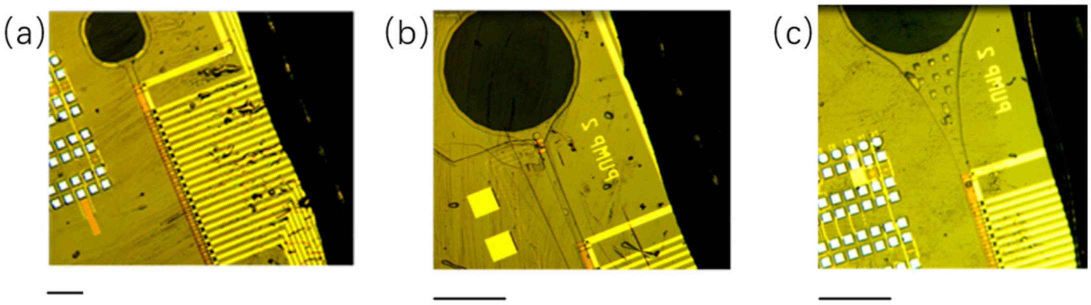

Figure S3. Three types of reservoirs. **(a)** Square shape with round corners **(b)** 1-mm diameter round shape; **(c)** Circular design with some cylinders. (Scale bar: 0.5 μm)

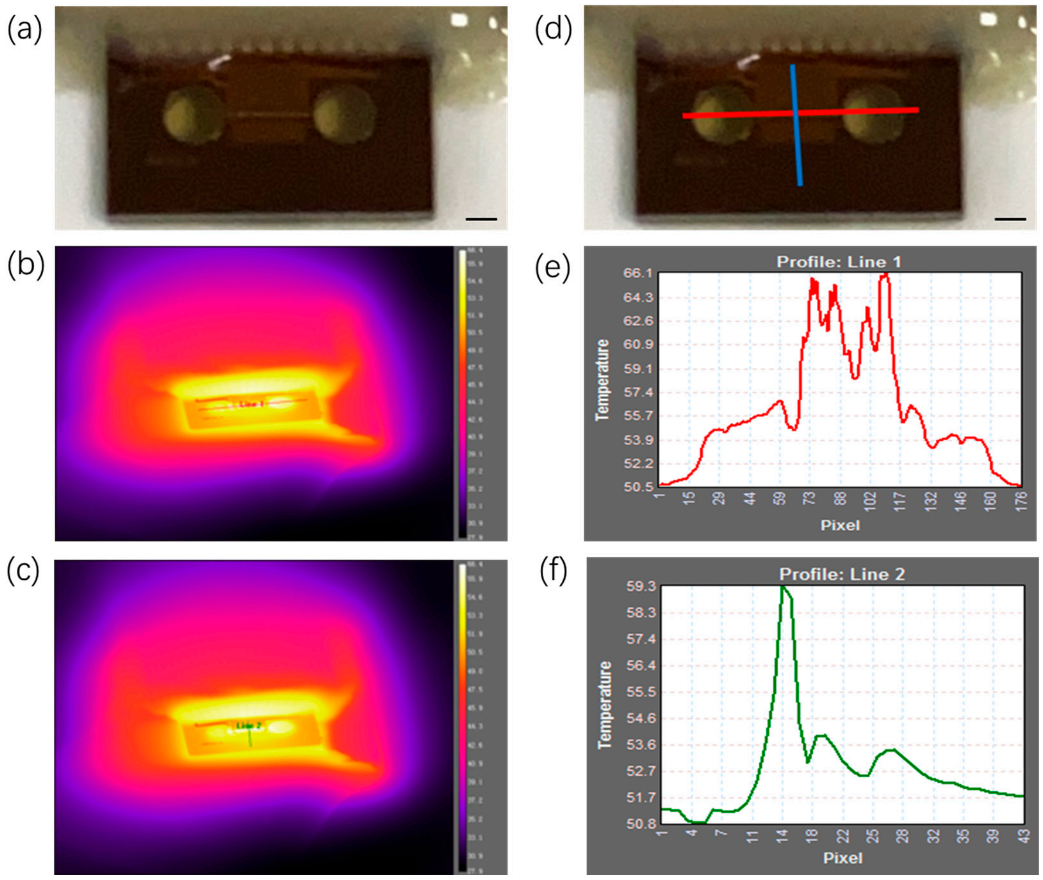

Figure S4. The temperature of the working micropump chip measured by thermal imager. (Scale bar: 0.5 µm) **(a)** The micropump entity; **(b)** The actual image taken by the thermal imager and where the temperature is read on the long axis; **(c)** The actual image taken by the thermal imager and where the temperature is read on the short axis; **(d)** The position where the chip the temperature is measured; **(e)** The temperature on the long axis; **(f)** The temperature on the short axis

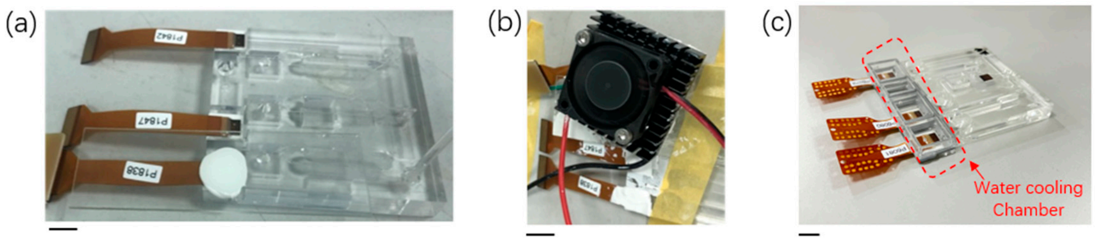

Figure S5. Different ways to control the temperature of the micropump. **(a)** The scheme of covering thermal grease; **(b)** Thermal grease and TEC cooling module; **c)** Water cooling module. (Scale bar: 24 mm)

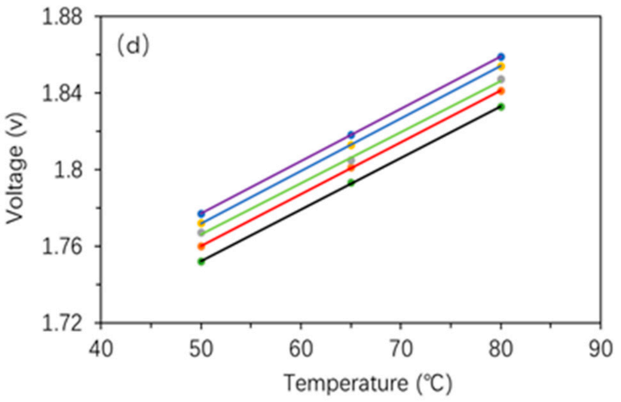

Figure S6. The linear relationship between temperature and voltage of thermometer

We would like to apply thermometer to measure the temperature of thermal bubble micropump when it was working. Before using the thermometer to accurately measure the temperatures of micropumps, we had to verify the sensitivity of the thermometer firstly (The voltage values revealed the detected temperatures in this thermometer). Therefore, we did some tests and obtained the different voltage values of thermometer on various temperatures as **Figure S6** shown (*five times of repeats*).
